# The Firepower of Work Craving: When Self-Control Is Burning under the Rubble of Self-Regulation

**DOI:** 10.1371/journal.pone.0169729

**Published:** 2017-01-09

**Authors:** Kamila Wojdylo, Nicola Baumann, Julius Kuhl

**Affiliations:** 1 Polish Academy of Sciences, Institute of Psychology, Warsaw, Poland; 2 Department of Psychology, University of Trier, Trier, Germany; 3 Department of Human Sciences, University of Osnabrück, Osnabrück, Germany; Universita degli Studi di Bologna, ITALY

## Abstract

Work craving theory addresses how work-addicted individuals direct great emotion-regulatory efforts to weave their addictive web of working. They crave work for two main emotional incentives: to overcompensate low self-worth and to escape (i.e., reduce) negative affect, which is strategically achieved through neurotic perfectionism and compulsive working. Work-addicted individuals’ strong persistence and self-discipline with respect to work-related activities suggest strong skills in volitional action control. However, their inability to disconnect from work implies low volitional skills. How can work-addicted individuals have poor and strong volitional skills at the same time? To answer this paradox, we elaborated on the relevance of two different volitional modes in work craving: self-regulation *(self-maintenance)* and self-control (*goal maintenance*). Four hypotheses were derived from Wojdylo’s work craving theory and Kuhl’s self-regulation theory: (*H1*) Work craving is associated with a combination of low self-regulation and high self-control. (*H2*) Work craving is associated with symptoms of psychological distress. (*H3*) Low self-regulation is associated with psychological distress symptoms. (*H4*) Work craving mediates the relationships between self-regulation deficits and psychological distress symptoms at high levels of self-control. Additionally, we aimed at supporting the discriminant validity of work craving with respect to work engagement by showing their different volitional underpinnings. Results of the two studies confirmed our hypotheses: whereas work craving was predicted by high self-control and low self-regulation and associated with higher psychological distress, work engagement was predicted by high self-regulation and high self-control and associated with lower symptoms of psychological distress. Furthermore, work styles mediated the relationship between volitional skills and symptoms of psychological distress. Based on these new insights, several suggestions for prevention and therapeutic interventions for work-addicted individuals are proposed.

## Introduction

Work-addicted individuals–so called workaholics–experience that they are only really “alive”, when working (e.g., [1], [2]). This situation indicates the essence of a craving desire for work. Intensive working is the main strategy available for work-addicted individuals to feel alive. Thus, work-addicted individuals’ sense of self is contingent on their persistent working. This suggests that work-addicted individuals have rather low skills in volitional action control. At the same time, however, work-addicted people’s high persistence and self-discipline when performing work-related activities suggest strong skills in volitional action control. To solve this paradox, we take an action control perspective on work craving that differentiates two types of volitional action control: self-regulation and self-control. We integrate both theoretical approaches in order to derive our hypotheses.

### Work Craving

Researchers have traditionally portrayed workaholism as a phenomenon that manifests almost entirely in obsessive-compulsive symptoms: obsessive thinking about work (intrusive thoughts that, for example, address the need to work, feeling that the job was not finishes, worry about not meeting a deadline) and a compulsive pattern of working behavior both behavioral and mental (e.g., [1], [2], [3]). By contrast, other scholars (e.g., [4]) argued that workaholism is an addiction disorder. Indeed, work-addicted individuals continue to engage in the same behavior (working) despite persistent adverse consequences. Moreover, in response to leisure time, they exhibit similar symptoms of withdrawal (including craving, restlessness, irritability, or exhaustion) as individuals who meet the criteria for addictive disorders, recognized by the APA (DSM-V, [5]). Like substance-addicted individuals, work-addicted individuals also develop a tolerance for the rewards of addictive means (work) manifested by a need for markedly increased amounts of their intoxicant (work) to achieve a desired effect [6]. There is evidence that behavioral addictions and substance addictions both share the same association with changes in the neural pathway of the reward system in the brain ([7], [8]) as well as with neurocognitive deficits, like those in executive functioning [9].

The theory of work craving, recently developed by Wojdylo [10], [11], tries to reconcile the seemingly contradictory positions regarding the obsessive-compulsive versus addictive nature of workaholism. Specifically, in contrast to previous theories of workaholism (e.g., [1], [12], [13]), it emphasizes the importance of a synthesis of obsessive-compulsive and *addictive* elements for better understanding the nature of work addiction. According to work craving theory ([10], [11]), work craving is defined as an *addictive disorder* (work addiction). Consequently, we use the terms “work craving” and “work addiction” interchangeably.

Work craving comprises both mechanisms typical for addiction: *positive reinforcement* as well as *negative reinforcement*. Specifically, the theory assumes that work-addicted persons become pathologically focused on *pursuing reward* from engaging in the behavior of compulsive working and neurotic standards: achieving positive outcomes such as self-worth, and social acceptance (positive reinforcement) and avoiding aversive outcomes such as negative emotions (negative reinforcement). Work-addicted individuals struggle with refraining from the behavior, experience intense craving, and have difficulty resisting; they have limited awareness of the problems that have emerged as a result of their behavioral addiction. Thus, according to the work craving theory, the phenomenon of workaholism is only dysfunctional. There is no functional form of workaholism (for a broader discussion see: [14]).

It should be noted that for convenience, we use the terms “work-cravers” and “work-addicted individuals” interchangeably for those individuals scoring relatively high on the Work Craving Scale (WCS), taking into account that the WCS has been analyzed as a continuous and not as a categorical variable (see: [11], [15], [16]). In contrast, we use the term “obsessive-compulsive workers” for individuals scoring relatively high on scales that narrowly measure “workaholism” as an obsessive-compulsive phenomenon. From a work craving perspective [10], there are two ways in which compulsive working may support addictive work processes. First, low self-worth and high negative emotions may stimulate individuals to crave an obsessive-compulsive style of working and to strategically persevere in such an action in order to gain overcompensation of low self-worth and sustain emotional escape from negative symptoms. Second, low self-worth may guide individuals to crave very high, unrealistic standards of achievement (neurotic perfectionism) and strategically persevere in such an action in order to gain overcompensation of low self-worth and sustain emotional escape from negative symptoms.

Wojdylo et al. [11] provided empirical evidence for the craving/addictive nature of workaholism. The findings showed that work craving comprises four empirically distinct dimensions: (1) obsessive-compulsive desire for work (obsessive thinking and compulsive working); two hedonic symptoms including (2) overcompensation of low self-worth resulting from overworking, and (3) escape from negative affect (emotional relief) and withdrawal symptoms (e.g., restlessness, irritability, or exhaustion); as well as the cognitive symptom (4) neurotic perfectionism.

There is also growing empirical evidence that work craving is embedded in a meaningful nomological network of related constructs and not conducive to health and well-being. For instance, work craving correlates positively with rumination, depression, and burnout as well as negatively with self-esteem and health [11], [15]. Recent results also confirm the detrimental effect of work craving on health in a longitudinal design [16]. It is therefore important to understand how work-addicted individuals regulate their unhealthy work style and what factors influence their “success” in doing so. A rather unexplored but important issue is how self-regulatory competencies are related with work craving (vs. work engagement) and, in turn, health problems. In studies where “workaholism” was measured only as an obsessive-compulsive phenomenon (and not as work addiction), subjective work experiences indicate that obsessive-compulsive workers and engaged workers both tend to work excessively hard (behavioral component) (e.g., [17]). However, their respective work styles serve different overarching purposes. Whereas obsessive-compulsive workers are driven to work by strong obsessive self-imposed demands they cannot resist (i.e., adopt external standards of self-worth and social approval without fully identifying with them; *introjected regulation*), engaged employees are working under the influence of intrinsic motivation, a positive connection with work, and the ability to deal with the demands of their jobs (i.e., engage in an activity for its own sake and act with a full sense of volition; *autonomous regulation*) [17].

According to work craving theory, the emotion regulatory strategies of work cravers (i.e., the desire for self-worth compensatory incentives through overworking and the desire to escape from negative emotions) imply that work craving should derive from low rather than high self-regulatory competencies. Indeed, Wojdylo, et al. [9] found that work cravers have deficits in self-regulating affect, whereas engaged workers have high competencies in self-regulating their emotions. Specifically, whereas work craving was related to a low ability to down-regulate negative emotions and to poor health, work engagement correlated with the high ability to up-regulate positive emotions and to good health [9]. Importantly, a longitudinal study confirmed that deficits in self-regulation of emotions influence work craving and, in turn, the symptoms of psychological distress [16].

These findings imply that work craving and work engagement may be associated with low versus high self-regulation competencies, respectively. This, in turn, inspires the question: How do work cravers manage to initiate and maintain their excessive work activities if they have poor volitional competencies? Their ability to focus on work-related goals suggests that work cravers also have strong volitional competencies.

## Self-Regulation and Self-Control

Kuhl’s theory of self-regulation provides a useful framework for understanding the volitional competencies of work cravers [18], [19], [20], [21]. According to self-regulation theory, the various volitional competencies individuals use to regulate their psychological functioning can be grouped into two basic modes of volition: self-regulation and self-control. *Self-regulation* is conceptualized as a “democratic” mode of volition in which individuals form goals in accordance with their needs and preferences (self-determination), regulate emotions in a context-adequate manner (self-motivation and self-relaxation), and flexibly resolve action-related conflicts. It is self-supportive and fosters an overview and integration of own needs, wishes, and goals. *Self-control*, in contrast, is conceptualized as an “authoritarian” mode of volition in which individuals maintain a specific goal by suppressing any tempting alternatives, even if these alternatives are self-congruent. It is self-suppressive, focused on goal maintenance and self-discipline, and supported by high planning abilities for specific action steps.

Whereas self-regulation operates by activating the reward system, identification with goals, and self-motivation through positive emotions (e.g., “I do this because it is important”), self-control operates by activating the punishment system, conscious and depletive efforts, and self-motivation via negative emotions (e.g., “If I don’t do this I will feel ashamed”) ([18], [19]). Consistent with this assumption, Kuhl and Fuhrmann [20] show that participants who report high self-regulation in daily life are more efficient in maintaining a healthy diet when rewarding themselves for dietary success (e.g., eating more broccoli and less ice cream) whereas participants high in self-control are more efficient when punishing themselves for dietary failures. This empirical dissociation supports the assumption that self-regulation and self-control are two distinct modes of volition.

Healthy psychological functioning requires both modes because self-regulation and self-control are adaptive under different conditions and have different (dis)advantages [22], [20]. Especially the overly strong use of self-control entails a trade-off between short-term benefits such as resistance to temptation and long-term costs such as alienation from own preferences and perseverance of stress [23], [24], [25]. Self-regulation, in contrast, entails positive short- and long-term effects because it promotes recovery from stress and correlates with fewer reports of psychopathology [24], [26]. In a study by Forstmeier and Rüddel [27], most of the self-regulation competencies correlated negatively with psychopathological measures such as depression as well as neuroticism, social inhibition, and excitability. However, to our knowledge, there is no systematic evaluation of the volitional skills of work-addicted individuals.

## Integrating Theories of Work Craving and Self-Regulation

The distinction between self-regulation and self-control helps to resolve the paradox that work cravers have both weak and strong volitional skills at the same time. On the one hand, it can be assumed that work cravers have poor emotion regulation skills and that working hard is their main way of coping with low self-worth and negative feelings (e.g., [11], [15]). Our studies showed that work cravers report difficulties with self-relaxation in daily life [15]. Other studies showed that obsessive-compulsive workers spend more time on work-related activities during the evening than non-obsessive-compulsive workers when experiencing negative emotions at the end of the workday [28], [29]. This suggests that work cravers have weak self-regulation (deficits in self-maintenance). On the other hand, it can be assumed that work cravers have high volitional skills because they manage to work excessively hard and show extreme drive in implementing work-related goals–albeit in an obsessive-compulsive manner [11], [15]. The studies showed that obsessive-compulsive workers tend to introject (rather than identify with) external requirements and perform actions in order to avoid guilt and anxiety [30]. This suggests that work cravers have *strong self-control* and strong activation of the associated punishment system *(competencies in goal maintenance)*. Biebrich and Kuhl [31] found the combination of low self-regulation and high self-control to yield coping responses that they consider to refer also to work addicted individuals: high action competence without inner security.

**Fig 1:**
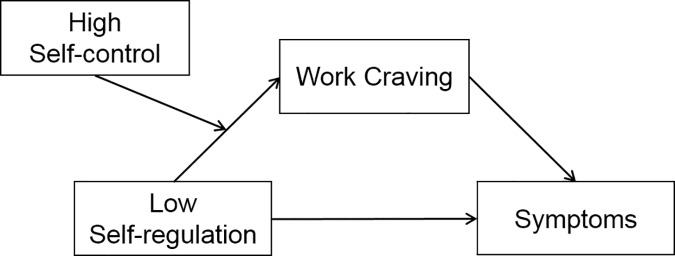
Conceptual model of the moderated mediation.

Taken together, we tested the following assumptions: (*H1*) Work craving is associated with low self-regulation and high self-control. (*H2*) Work craving is associated with symptoms of psychological distress. (*H3*) Low self-regulation is associated with symptoms of psychological distress. Because of the mixed effects of self-control—positive short-term effects as well as negative long-term effects of (too much) self-control [23]—, we did not make a prediction about the relationship between self-control and distress symptoms. (*H4*) Consistent with previous findings of Wojdylo and Baumann et al. [15] on work craving, we expected work craving to mediate the relationship between volitional skills and distress symptoms. As depicted in Fig 1, we expected a moderated mediation: Work craving mediates the relationship between low self-regulation and distress symptoms at high levels of self-control. We tested our hypotheses in two studies that are part of the Work Craving International Project (WCIP), a cohort research endeavor of Poland and Germany. The goal of the WCIP is to study the new conceptualization of workaholism as work craving and its underlying personality antecedents.

## Study 1

### Participants

Researchers recruited 291 participants who were Polish employees (174 women = 59.79%) in different Polish companies (e.g., employees of business and information technology services, employees of the electric company, teachers, office and administrative workers, etc.). In each of the respective participating companies, about 90–95% of the employees participated. Their mean age was 41 years (range 22–63 years). Most participants held nonmanagerial (77.3%) rather than managerial (22.7%) positions; 75.6% of participants had completed higher education, 22.3% secondary education, and 2.1% vocational education.

### Ethics Statement

We carried out the study procedures in accordance with the Declaration of Helsinki.

We collected information anonymously and did not have access to identifying participant information. These project studies received approval from the German ethics committee before commencing with the investigations (Aufsichts- und Dienstleistungsdirektion Rheinland-Pfalz: ADD 51 111-32/129-12; Landesbeauftrage für Datenschutz und Informationsfreiheit Rheinland-Pfalz: LfD RLP 6.08.22.001:0363). The ethics committee in Poland confirmed that the study met applicable standards for the ethics of experimentation and research integrity.

### Materials

#### Self-Regulation and Self-Control

We used the Polish version of the Volitional Components Inventory (VCI; [20] to assess self-regulation and self-control. The self-regulation scale consists of 12 items (Cronbach’s α = .89, in the present study) covering self-determination (*“I feel that most of the time I really want to do the things I do”*), self-motivation (*“When my enthusiasm drops off*, *I know exactly how to motivate myself again”*), and self-relaxation (*“I know exactly how to decrease my anxiety”*). The self-control scale consists of 8 items (Cronbach’s α = .83, in the present study) covering planning abilities (*“Before I approach a new task*, *I usually make a plan first”*) and anxious goal-orientation (*“In order to motivate myself I often imagine what would happen if I didn't finish the task on time”*). Participants rate the extent to which each statement applies to them on a 4-point scale ranging from 1 (*not at all*) to 4 (*completely*). Kuhl and Fuhrmann [20] as well as Fröhlich and Kuhl [22] reported extensive research on the reliability and validity of the scales.

#### Work craving

In order to measure work craving, the Polish version of the Work Craving Scale (WCS; [10]) was administered. The questionnaire consists of 28 items (Cronbach’s α = .95, in the present study) covering an obsessive-compulsive desire for work (*“My desire for work overpowers me”*), overcompensation of low self-worth resulting from overworking, (*“Overworking makes me feel important”*), escape from negative affect (emotional relief) and withdrawal symptoms (*“I can relax only if I’m working hard”*), and neurotic perfectionism (*“Even though I perform a task very carefully*, *I feel that it is done not correctly enough*”). Participants indicate to what extent they (dis)agree with each statement on a 7-point scale ranging from 1 (*I completely do not agree)* to 7 (*I completely agree)*. For extensive research on the reliability and validity of the scales, see Wojdylo et al. [10].

#### Psychological distress symptoms

To assess mental health status we used the Polish version of the General Health Questionnaire (GHQ-28; [32]). It consists of 28 items (Cronbach’s α = .91, in the present study) covering somatic symptoms (*“Have you recently felt that you are ill*?*”*), anxiety and insomnia (*“Have you recently lost much sleep over worry*?*”*), social dysfunction (*“Have you recently been taking longer over the things you do*?*”*), and severe depression (*“Have you recently felt that life is not worth living*?*”*). Participants rated items on four-point scales ranging from 1 to 4 with slightly different labels across items. Higher scores indicate lower general mental health and higher psychological distress.

### Design and Procedure

Researchers recruited participants via an e-mail that was distributed throughout the companies that had given informed consent to take part in the study. The e-mail included an Internet link to a website that could be accessed by those workers interested in participating in an online survey on work behaviors and health. Data are based on anonymized self-report questionnaires administered during working hours (S1 File). The participants were not reimbursed for their participation.

### Results

#### Descriptive statistics and correlations

Table 1 presents the means, standard deviations, and correlations among the study variables in Study 1. All correlations were in the expected direction. Consistent with *H1*, work craving exhibited a significantly negative correlation with self-regulation and a significantly positive correlation with self-control. Consistent with *H2*, a significantly positive correlation was found between work craving and symptoms of psychological distress. Consistent with *H3*, we found a significantly negative correlation of self-regulation with psychological distress. Self-control did not significantly correlate with psychological distress symptoms. There were no significant relationships between work craving and age or gender.

**Table 1:** *Correlations*, *Means*, *and Standard Deviations of Variables in Studies 1 (*N *= 291; upper right) and 2 (*N *= 272; lower left)*.

	(1)	(2)	(3)	(4)	(5)	(6)	(7) ^a^	(8) ^a^	*M*	*SD*
(1) Self-regulation		.43 ***	-.12 *		-.44 ***	.07	.01	.12 *	2.59	.53
(2) Self-control	.32 ***		.12 *		-.11	.13 *	.01	.06	2.71	.59
(3) Work Craving	-.21 ***	.12			.23 ***	-.02	.01	-.12 *	2.82	.92
(4) Work Engagement	.33 ***	.20 ***	.30 ***							
(5) Distress Symptoms	-.47 ***	-.06	.24 ***	-.38 ***		.01	.16 **	-.15 *	1.94	.40
(6) Age	.18 **	.14 *	.11	.49 ***	-.12 *		.01	.21 ***	40.56	9.71
(7) Gender ^a^	-.10	.07	-.10	.02	.11	.04		-.29 ***		
(8) Management Position ^a^	.22 *	.10	.07	.41 ***	-.11	.48 ***	.08			
*M*	2.49	2.61	2.70	4.35	2.04	29.93				
*SD*	.51	.63	1.01	1.41	.48	6.91				

*Note*. Gender: 1 = male, 2 = female. Management position: 1 = no, 2 = yes.

^a^ We calculated Spearman-Rho (instead of Pearson) correlations for the dichotomous variables.

* *p* < .05.

** *p* < .01.

*** *p* < .001.

#### Moderated mediation analysis

To test whether work craving mediated the negative relationship between self-regulation and symptoms of psychological distress at high levels of self-control (*H4*), we conducted a moderated mediation analysis with 5,000 bootstrap resamples using the SPSS macro (Model 8) described by Hayes [33]. Using this procedure, we computed a point estimate and a 95% confidence interval (CI) for the moderated mediation effect. We entered self-regulation as an independent variable, self-control as a moderator, work craving as a mediator, and psychological distress as an outcome variable.

#### Work craving

**Fig 2:**
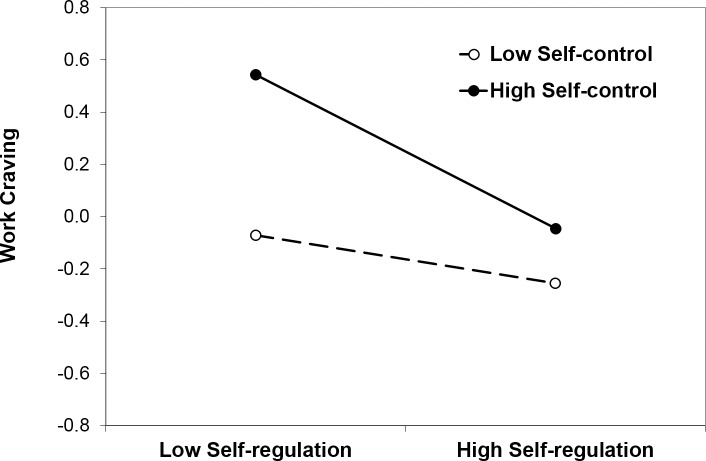
Work craving as a function of self-regulation and self-control in Study 1. Insert here.

In the model testing work craving as a dependent variable (see Table 2), there were significant main effects of self-regulation and self-control indicating that self-regulation was associated with lower and self-control with higher work craving. Consistent with expectations, the self-regulation x self-control interaction was significant. In Fig 2, the interaction effect is illustrated for values of *M* ± 1 *SD* for both predictor variables. At low levels of self-control, work craving was low irrespective of the level of self-regulation (*ß* = -.09, *t* = -1.14, *ns*). At high levels of self-control, in contrast, lower self-regulation was associated with higher work craving (*ß* = -.29, *t* = -3.96, *p* < .001). Similarly, at high levels of self-regulation, work craving was low irrespective of the level of self-control (*ß* = .11, *t* = 1.35, *ns*). At low levels of self-regulation, in contrast, higher self-control was associated with higher work craving (*ß* = .31, *t* = 3.99, *p* < .001). Findings are consistent with our hypothesis that work craving is associated with a combination of low self-regulation and high self-control.

**Table 2:** *Summary of Direct Effects of Self-Regulation and Self-Control on Work Styles in Studies 1 (*N *= 291) and 2 (*N *= 272)*.

	Study 1: Work Craving				Study 2: Work Craving				Study 2: Work Engagement			
	*R*^2^	*β*	*t*		*R*^2^	*β*	*t*		*R*^2^	*β*	*t*	
	.06 ***				.11 ***				.13 ***			
Constant		.04	.72			.05	.76			.03	.58	
Self-regulation (SR)		-.19	-3.04	**		-.27	-4.36	***		.30	5.04	***
Self-control (SC)		.21	3.26	**		.20	3.30	**		.10	1.68	
SR x SC		-.10	-2.22	*		-.14	-2.55	*		-.11	-1.97	*

* *p* < .05.

** *p* < .01.

*** *p* < .001.

#### Psychological distress symptoms

In the model testing symptoms as a dependent variable (see Table 3), there were significant main effects of self-regulation and work craving on symptoms, indicating that self-regulation was associated with lower and work craving with higher symptoms. Self-control had no main or interaction effects on symptoms when controlling for work craving.

**Table 3:** *Summary of Direct Effects of Self-Regulation*, *Self-Control*, *and Work Styles on Psychological Distress Symptoms in Studies 1 (*N *= 291) and 2 (*N *= 272)*.

	Study 1: Distress Symptoms				Study 2: Distress Symptoms			
	*R*^2^	*β*	*t*		*R*^2^	*β*	*t*	
	.23 ***				.36 ***			
Constant		-.00	-.05			-.02	-.45	
Work Craving		.17	3.20	**		.28	4.98	***
Work Engagement						-.37	-6.44	***
Self-Regulation (SR)		-.45	-7.72	***		-.33	-5.61	***
Self-Control (SC)		.07	1.14			.09	1.72	
SR x SC		.01	.17			.07	1.51	

** *p* < .01.

*** *p* < .001.

The significance of the indirect effect of low self-regulation through work craving on symptoms was tested across low, moderate, and high levels of self-control with bootstrapped errors and 95% confidence intervals (CIs). Consistent with expectations (*H4*), the indirect effect of low self-regulation through work craving on symptoms was significant at moderate and high levels of self-control (because the limits of the 95% confidence interval did not include zero) but not at low levels of self-control (see Table 4).

**Table 4:** *Summary of Conditional Indirect Effects of Self-regulation (SR) through Work Styles on Psychological Distress Symptoms at Values of M ± 1 SD of Self-control (SC) as well as Indirect Effects of the SR x SC Interaction through Work Styles on Psychological Distress Symptoms in Studies 1 (*N *= 291) and 2 (*N *= 272)*.

	Study 1: Distress Symptoms				Study 2: Distress Symptoms			
	*B*	*Boot SE*	*Boot LLCI*	*Boot ULCI*	*B*	*Boot SE*	*Boot LLCI*	*Boot ULCI*
SR → Work Craving → Symptoms at Values of								
Low Self-control	-.02	.02	-.06	.01	-.04	.02	-.09	.01
Moderate Self-control	-.03	.02	-.09	-.01	-.07	.02	-.13	-.04
High Self-control	-.05	.03	-.12	-.02	-.11	.03	-.19	-.06
SR x SC → Work Craving → Symptoms	-.02	.01	-.05	-.01	-.04	.02	-.08	-.02
SR → Work Engagement → Symptoms at Values of								
Low Self-control					-.15	.04	-.24	-.09
Moderate Self-control					-.11	.03	-.18	-.06
High Self-control					-.07	.03	-.15	-.02
SR x SC → Work Engagement → Symptoms					.03	.02	.01	.08

*Note*. LLCI = Lower Limit Confidence Interval; ULCI = Upper Limit Confidence Interval.

The indirect effect of the self-regulation x self-control interaction through work craving on symptoms was significant. This finding indicates that a combination of low self-regulation and high self-control may be problematic because it fosters work craving and, in turn, symptoms of psychological distress (see Table 4).

## Study 2

In Study 2, we aimed at replicating our findings in a different sample of workers (e.g., managerial, administrative, and clerical employees in areas such as banking services, data analysis, office administration, etc.). In addition, we wanted to support the discriminant validity of work craving with respect to work engagement by showing their different volitional underpinnings. As mentioned earlier, work craving and work engagement have distinct regulatory mechanisms and outcomes: an obsessive-compulsive drive versus intrinsic motivation [17], low versus high self-regulation [15], and low versus high psychological health [11], [15]. These findings imply that work engagement is related to high self-regulation competencies. Whereas the sole reliance of work craving is on self-control rather than self-regulation, we expected work engagement to be associated with high abilities in both self-regulation and self-control. Furthermore, we expected work engagement to be negatively associated with symptoms of psychological distress. We also expected work engagement to mediate the relationship between volitional skills and distress symptoms.

### Participants

Researchers recruited 272 participants who were employees (158 women = 58.09%) in various Polish companies (e.g., banking services employees, data analysts, office workers, economists, etc.). In each of the respective participating companies, about 90–95% of the employees participated. Their mean age was 30 years (range 21–52 years). Most participants were employed in nonmanagerial (82%) rather than managerial (18%) positions; 71.3% of participants had completed higher education and 28.7% secondary education.

### Materials

We used the same measures for self-regulation (Cronbach’s α = .86, in the present study), self-control (Cronbach’s α = .82, in the present study), work craving (Cronbach’s α = .95, in the present study), and psychological distress (Cronbach’s α = .88, in the present study) as in Study 1.

### Work engagement

To assess work engagement, we used the Polish version of the Utrecht Work Engagement Scale (UWES; [34]). The measure consists of 17 items (Cronbach’s α = .94, in the present study) covering vigor (*“At my work*, *I feel bursting with energy”)*, dedication, (*“I find the work that I do full of meaning and purpose”)*, and absorption (*“When I am working*, *I forget everything else around me”)*. Participants rated items on a 7-point frequency scale ranging from 1 (*never*) to 7 (*each day*). Higher scores indicator higher work engagement.

### Design and Procedure

We used the same strategy to recruit participants as in Study 1 (S2 File).

### Results

#### Descriptive statistics and correlations

Table 1 presents the means, standard deviations, and correlations among the study variables in Study 2. All correlations were in the expected direction. Consistent with *H1*, work craving exhibited a significant, negative correlation with self-regulation and a (marginally) significant and positive correlation with self-control. Work engagement, in contrast, was significantly and positively correlated with self-regulation and self-control. Consistent with *H2*, a positive correlation was found between work craving and psychological distress symptoms, and a negative correlation was found between work engagement and psychological distress symptoms. Consistent with *H3*, self-regulation correlated negatively with distress symptoms. There was no correlation between self-control and distress symptoms. There were no significant relationships between work craving and age or gender.

#### Moderated mediation analysis

We conducted the same analysis (SPSS macro Model 8; [33]) as in Study 1 with the following change: In addition to work craving, we entered work engagement as a mediator into the analysis.

#### Work craving

In the model testing work craving as a dependent variable (see Table 2), there were significant main effects of self-regulation and self-control, indicating that self-regulation was associated with lower and self-control with higher work craving. Consistent with expectations and findings in Study 1, the self-regulation x self-control interaction was significant. At low levels of self-control, work craving was low and not related to self-regulation (*ß* = -.13, *t* = -1.45, *ns*). At high levels of self-control, in contrast, lower self-regulation was associated with higher work craving (*ß* = -.41, *t* = -5.05, *p* < .001). Similarly, at high levels of self-regulation, work craving was low and not related to self-control (*ß* = .06, *t* = .71, *ns*). At low levels of self-regulation, in contrast, higher self-control was associated with higher work craving (*ß* = .34, *t* = 4.21, *p* < .001). Findings are consistent with our hypothesis that work craving is associated with a combination of low self-regulation and high self-control.

#### Work engagement

**Fig 3:**
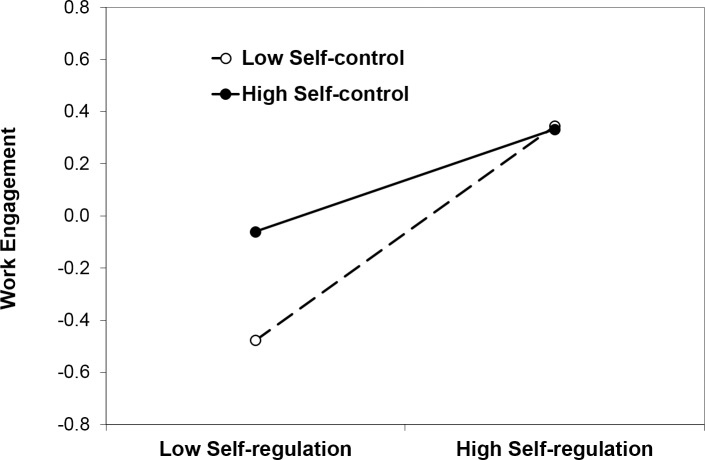
Work engagement as a function of self-regulation and self-control in Study 2.

In the model testing work engagement as a dependent variable (see Table 2), there was a significant main effect of self-regulation indicating that higher self-regulation was associated with higher work engagement. In addition, there was a significant self-regulation x self-control interaction. As illustrated in Fig 3, at low levels of self-control, lower self-regulation was associated with significantly lower work engagement (*ß* = .41, *t* = 4.76, *p* < .001). This relationship was less pronounced but still significant at high levels of self-control (*ß* = .20, *t* = 2.43, *p* < .02). Similarly, at high levels of self-regulation, work engagement was high irrespective of the level of self-control (*ß* = -.01, *t* = 0.07, *ns*). At low levels of self-regulation, in contrast, work engagement was low but reached moderate levels with higher self-control (*ß* = .21, *t* = 2.56, *p* < .02). The finding is consistent with the assumption that work engagement has different volitional underpinnings than work craving.

As summarized in Table 5, self-regulation is a necessary and sufficient volitional competency for work engagement. Whether or not employees are able to exert self-control does not affect their level of work engagement as long as they are able to self-regulate emotions. In contrast, sole reliance on self-control in the absence of self-regulation is associated with work craving. Finally, a combination of low self-regulation and low self-control is associated with low levels of both work styles and may be characterized as low work involvement.

**Table 5:** Summary of Relationships between Volitional Competencies and Work Styles.

	Self-regulation (“inner democracy”)	
	Low	High
Self-control (“inner dictatorship”)		
Low	Low Work Involvement	Work Engagement
High	Work Craving	Work Engagement

#### Psychological distress symptoms

In the model testing symptoms as a dependent variable (see Table 3), there were significant main effects of work craving, work engagement, and self-regulation on symptoms, indicating that work craving was associated with higher symptoms whereas work engagement and self-regulation were associated with lower symptoms. Self-control had no main or interaction effects on symptoms when controlling for work styles.

The significance of the indirect effect of low self-regulation through work styles on symptoms was tested across low, moderate, and high levels of self-control with bootstrapped errors and 95% confidence intervals (CIs). Consistent with expectations (*H4*), the indirect effect of low self-regulation on symptoms through increased work craving was significant at moderate and high levels of self-control (because the limits of the 95% confidence interval did not include zero) but not at low levels of self-control (see Table 4). Furthermore, the indirect effect of low self-regulation on symptoms through reduced work engagement was significant across all levels of self-control.

The indirect effects of the self-regulation x self-control interaction through work styles on symptoms were significant for both variables, work craving and work engagement (see Table 4). Findings indicate that two different mechanisms partially mediated the relationship between low self-regulation and symptoms. First, in conjunction with high self-control, low self-regulation aggravated the unhealthy work style of work craving. Second, in conjunction with low self-control, low self-regulation undermined the healthy work style of work engagement.

## Discussion

The present studies aimed at contributing to a better understanding of workaholism: specifically, its addictive nature and self-regulatory mechanisms. First, using Wojdylo’s theory of work craving [10] we outlined workaholism as a clearly pathological work style and extended it by hedonic and cognitive components to define work craving. It encompasses not only compulsive behaviors but also emotion-regulatory components characteristic of addiction disorders (i.e., a desire for self-worth compensatory incentives and relief from negative emotions through neurotic perfectionism). Second, we applied Kuhl’s [18], [19] theory of self-regulation to differentiate two modes of volition: self-regulation (i.e., an “inner democracy” oriented at self-maintenance and integration of multiple needs, goals, and preferences) and self-control (i.e., an “inner dictatorship” oriented at goal maintenance and perseverance of a single goal). Finally, we integrated both theories and predicted work craving to be associated with a combination of low self-regulation and high self-control.

In two samples of Polish workers (total of 563 participants), we found work craving to be associated with the expected combination: work cravers have a low ability to form self-congruent goals (low self-regulation) but a high ability to maintain goals and suppress tempting alternatives which is conducive to successful goal striving (high self-control). However, even successful goal striving does not promote well-being if goals are not integrated into the self and do not satisfy personal needs [35]. Koole et al. [36] refer to conditions in which people are chronically locked into self-control as *ego fixation* and review its role in alienation from emotional preferences, rigidity, overcontrol, rumination, and psychosomatic problems [35], [37], [23], [25]. Consistent with this negative view on chronic self-control, our findings showed work craving to be associated with symptoms of psychological distress symptoms. Furthermore, work craving mediated the relationship between self-regulation deficits and symptoms at moderate and high levels of self-control. Thus, strong self-control did not buffer against the negative effects of weak self-regulation because it fueled an unhealthy work style. Metaphorically speaking, the dominance of self-control in the personality (“when self-control is burning”), together with deficits of self-regulation (“under the rubble of self-regulation”) forms the “inflammatory,” destructive mechanism of work craving resulting in reduced health.

In light of these findings, one may wonder why work cravers keep on going. Previous findings show that obsessive-compulsive workers are avoidance motivated and try to prevent failure, guilt, shame, and anxiety [13], [38], [39]. At the same time, avoidance motivation is a strong source of self-control [20], [19]. Based on our findings, it can be concluded that work cravers’ strong self-control skills make them highly efficient in avoiding negative emotions. This may explain why they remain so persistent in their work activities and why it is so difficult to change their unhealthy behavior (cf. [11], [15], [16]). Furthermore, high self-control makes people suppress intuitive (dis)likes and bodily responses that may serve as “somatic markers” [40] to guide people away from potentially dangerous and self-incongruent choices or toward successful and self-congruent options [36]. On the one hand, this may lead people to become alienated from their intuitive dislike for aversive experiences. It may explain the obstinate persistence of work-addicted individuals’ destructive work habits despite negative consequences (e.g., distress symptoms, exhaustion). On the other hand, the lack of inner reference may explain why work cravers are not even satisfied with successful results, set neurotically high standards, and apply a social reference norm [13]. Taken together, work cravers are characterized by their high action competencies without inner security (cf. [31]).

It is noteworthy that our findings were obtained in two heterogeneous general population samples: workers with different occupations and from different work environments. Work craving theory does not specify work environments or job characteristics. Instead, work craving is assumed to develop as a function of personality and may be utilized as a coping mechanism in nonwork contexts, for example, in college or after retirement. Items may have to be adjusted to allow a valid assessment of work craving in such contexts. Thus, work craving theory takes a personality perspective on work addiction.

In support of the discriminant validity of work craving, we found work engagement to have a completely different profile of volitional competencies: high self-regulation and high self-control. Furthermore, higher work engagement was associated with lower psychological distress and mediated the relationship between self-regulation competencies and health. Thus, work engagement is clearly a healthy work style. Engaged workers’ self-regulatory ability to integrate the multitude of personal needs, wishes, and goals may also contribute to successful work-life balance [41]. Taken together, engaged workers are characterized by high action competencies combined with inner security (cf. [31]).

Note that self-regulation and self-control show a significant positive correlation in our studies. This is because both are volitional competencies. Functionally distinct dimensions can correlate to the extent that they cooperate under certain conditions. In healthy individuals, self-control and self-regulation should cooperate whenever this seems reasonable, for example, when it makes sense to invest impulse control (as an example of self-control) after having made a self-congruent decision (as an example of self-regulation). Work engagement seems to indicate this type of healthy cooperation. Nevertheless, self-regulation and self-control are distinct volitional competencies because they may dissociate: one competency may be available in excess while the other is deficient. Work craving seems to indicate this type of dissociation and one-sided volitional action control (excessive self-control and deficient self-regulation).

## Limitations and Future Perspectives

Our study has several limitations that can be addressed in future research. First, our findings are cross-sectional and do not allow us to draw causal conclusions. However, self-regulation and self-control are relatively stable dispositions that develop from early childhood onwards [18], [19]. Therefore, it is unlikely that the scores on the volitional measures were caused by work styles or health. In contrast, the causal direction of the association between health and work styles is less clear. However, in a longitudinal design, Wojdylo et al. [16] showed that work craving predicted health decrements whereas health did not predict changes in work craving.

Second, our outcomes are merely self-reports of psychological distress symptoms. In future studies, it would be informative to broaden the network of possible outcomes (e.g., job performance, work-life balance) and to extend measures to less obtrusive data (external ratings, behavioral indicators). Third, we have concentrated on a personality perspective in our studies. It would be informative to assess work environment factors in order to explore their role in strengthening or ameliorating volitional competencies and/or work styles. For instance, employees’ self-control and work craving may be fostered in a company where the policy and its managers focus on the task and time pressure. In contrast, a company that values benevolence may employ more autonomy-supportive managers who foster employees’ self-regulation and work engagement. Future studies can verify if a more supportive work environment may compensate employee’s self-regulation deficits and function as a moderator at different positions in the model depicted in Fig 1.

Finally, our findings have important practical implications. Forstmeier and Rüddel [27] have indicated that improving volitional competencies is crucial for the efficacy of treatment of psychosomatic symptoms in psychotherapy. Thus, it can be concluded that improving volitional competencies is one of the most relevant issues in the prevention and treatment of work addiction and its effects on psychosomatic symptoms. Our findings highlight the importance of differentiating between self-regulation and self-control to understand the specific problems and resources of work-addicted individuals. Intervention programs should focus especially on increasing self-regulation competencies in employees [42] such as: (a) generating many alternative ways to reach a goal, (b) expanding attention to the multitude of personal needs, wishes, and goals, (c) feeling and differentiating own emotions and body signals, and (d) training self-motivation and self-relaxation. Furthermore, organizations should encourage an organizational climate and leadership style that supports self-regulation and autonomy in their staff to prevent work craving and promote employees’ health [43], [44].

## Supporting Information

S1 File Study 1 Dataset. (XLS)

S2 File Study 2 Dataset. (XLS)
